# Assembly of Cell-Free Synthesized Ion Channel Molecules in Artificial Lipid Bilayer Observed by Atomic Force Microscopy

**DOI:** 10.3390/membranes13110854

**Published:** 2023-10-25

**Authors:** Melvin Wei Shern Goh, Yuzuru Tozawa, Ryugo Tero

**Affiliations:** 1 Department of Applied Chemistry and Life Science, Toyohashi University of Technology, Toyohashi 441-8580, Japan; 2 Graduate School of Science and Engineering, Saitama University, Saitama 338-8570, Japan; tozawa@mail.saitama-u.ac.jp

**Keywords:** supported lipid bilayer, cell-free expression, ion channel, hERG, k_v_11.1, atomic force microscopy

## Abstract

Artificial lipid bilayer systems, such as vesicles, black membranes, and supported lipid bilayers (SLBs), are valuable platforms for studying ion channels at the molecular level. The reconstitution of the ion channels in an active form is a crucial process in studies using artificial lipid bilayer systems. In this study, we investigated the assembly of the human *ether-a-go-go-*related gene (hERG) channel prepared in a cell-free synthesis system. AFM topographies revealed the presence of protrusions with a uniform size in the entire SLB that was prepared with the proteoliposomes (PLs) incorporating the cell-free-synthesized hERG channel. We attributed the protrusions to hERG channel monomers, taking into consideration the AFM tip size, and identified assembled structures of the monomer that exhibited dimeric, trimeric, and tetrameric-like arrangements. We observed molecular images of the functional hERG channel reconstituted in a lipid bilayer membrane using AFM and quantitatively evaluated the association state of the cell-free synthesized hERG channel.

## 1. Introduction

Cell membranes are highly complex matrices composed of a vast array of macromolecules, in particular, a wide range of lipids and membrane proteins [1,2]. The transportation of ions, signals, and energy into and out of cells is conducted through membrane proteins, and the two-dimensional organization of membrane proteins and lipids plays an essential role in these reactions [3,4,5,6]. Ion channels are membrane proteins that regulate ion fluxes across the cell membranes for neuronal signal transduction, control of osmotic pressure, and the generation of action potential (AP). Malfunction of ion channels is associated with various diseases, and thus, they are regarded as an important subject in the fields of medicine and drug discovery [7,8,9]. In multimeric ion channels, the assembly of homologous structural units results in a small pore at the central axis, serving as an ion-conducting passage. Artificial lipid bilayer systems, such as vesicles, black membranes, and supported lipid bilayers (SLBs), are valuable platforms for the study of ion channels at the molecular level [10,11].

The human *ether-a-go-go*-related gene (hERG) channel [12,13,14,15,16] is a voltage-dependent potassium (K_v_) channel encoded by KCNH2. It is primarily found in neurons and cardiac cells, and its function is well understood in the heart. The hERG channel is responsible for mediating the rapid delayed rectifier K^+^ current, which makes a major contribution to the repolarization phase of cardiac AP. During the repolarization phase, the hERG channel quickly reopens and recovers from the inactivated state, facilitating AP termination, and maintains the QT interval on the electrocardiogram. Unintended side effects on the hERG channel due to off-target toxicity of clinical drugs causes serious cardiac arrhythmia [13,14,15]. Therefore, structural observation of the hERG channel is crucially important for expediting the drug discovery process by shedding light on the underlying inhibition mechanism caused by a wide variety of chemically diverse drugs [17]. Recently, the high-resolution structure of the pore domain of the hERG channel was revealed at a resolution of 3.8 Å using cryo-electron microscopy [18]. Wang and Mackinnon constructed a truncated hERG channel with two segments being deleted (141-350 and 871-1005) to prevent molecular aggregation and increase molecular stability [18]. The truncated construct contains 814 amino acid residues, while a full-length hERG channel contains 1159 amino acid residues.

Recent advances in cell-free synthesis technology have stimulated profound interest in the field of membrane protein research using artificial cell membrane systems [19,20,21,22]. Cell-free synthesis systems enable the direct in vitro production of pure target proteins with the full length of amino acid residues derived from a protein-encoding plasmid, without the need for cell culturing and protein purification. These systems are based on various sources, including cell extracts such as those from *E. coli* and wheat-germ [23,24], as well as recombinant proteins [25]. Initially, the method is developed for water-soluble proteins and subsequently is applied to membrane proteins [19,20,21,22]. By conducting cell-free synthesis of membrane proteins in the presence of artificial lipid bilayers, e.g., lipid vesicles (also referred to as liposomes) and nanodiscs, under appropriate conditions, the proteins are reconstituted into the lipid bilayer during the synthesis process and obtained as proteoliposomes (PLs). The next critical phase involves the development of experimental methods to investigate whether the cell-free synthesized membrane proteins consistently reproduce their structures and functions in native membranes. This step is essential for validating the utility of the cell-free synthesized proteins in artificial biomembrane systems.

Recently, Tadaki et al. successfully recorded the activities of the cell-free-synthesized full-length hERG channel by the free-standing bilayer lipid membrane (BLM) method using a microfabricated silicon chip with a nanotapered aperture [26]. The ternary BLM consisting of egg-derived phosphatidylcholine (PC), egg-derived phosphatidylethanolamine (PE), and cholesterol (Chol) in the study is capable of retaining the activity of various ion channels [27,28,29]. The PL incorporating the hERG channel was prepared using a wheat germ cell-free translation system by expressing the hERG channel in the presence of PC+PE+Chol vesicles. The single-channel current of the hERG channel appeared after the addition of the hERG-expressed PL to the BLM and was inhibited with astemizole, which is a representative inhibitor for the hERG channel [26]. Recent studies on ternary PC+PE+Chol-SLBs revealed that the fusion of PLs is promoted by microdomains existing in the PC+PE+Chol bilayer [30,31], and that the microdomains are rich in polyunsaturated lipids [32,33].

In this study, our aim is to observe the molecular structure and association states of the hERG channel in the bilayer membrane by atomic force microscopy (AFM) using the SLB system [34,35,36,37]. Even though the single-channel current measurement of the cell-free synthesized hERG channel was achieved [26], the throughput of the channel recording is still a subject for improvement [28,38]. The cell-free synthesis system provides full-length hERG channel monomers with high purity, but the efficiency in assembling these monomers into the functional tetrameric state remains unknown. AFM is a powerful technique that enables the observation of membrane proteins at a single-molecule level [39,40,41,42,43]. A methodology for evaluating the association states of cell-free synthesized membrane proteins is valuable for improving the efficiency of cell-free synthesis and the reconstitution of membrane proteins in artificial lipid membrane systems such as the BLM and SLB.

## 2. Materials and Methods

The phospholipid reagent L-α-phosphatidylcholine (from egg) (PC) and L-α-phosphatidylethanolamine (transphosphatidylated (egg)) (PE) were purchased from Avanti Polar Lipids (Alabaster, AL, USA). 1,2-bis-(4,4-difluoro-5,7-dimethyl-4-bora-3a,4a-diaza-*s*-indacene-3-undecanoyl)-*sn*-glycero-3-phosphocholine (Bis-Bodipy-FL-PC, Ex/Em: 500/510 nm) was purchased from Thermo Fisher Scientific (Waltham, MA, USA). These phospholipid agents were used as received, without further purification. Chol was purchased from Wako Pure Chemicals (Osaka, Japan), and recrystallized three times from methanol prior to use. The chloroform solutions of PC, PE, and Chol were mixed at a ratio of PC:PE:Chol = 7:1:2 (*w*/*w*) (molar ratio of 0.58:0.09:0.33) in a clean glass vial. The mixed solution was dried using a stream of nitrogen, and the dried lipid film was sonicated in a buffer solution (120 mM KCl and 10 mM HEPES/KOH (pH 7.2)) for an hour to prepare unilamellar vesicles. The PC+PE+Chol vesicles were freshly prepared immediately prior to use. 

**Figure 1:**
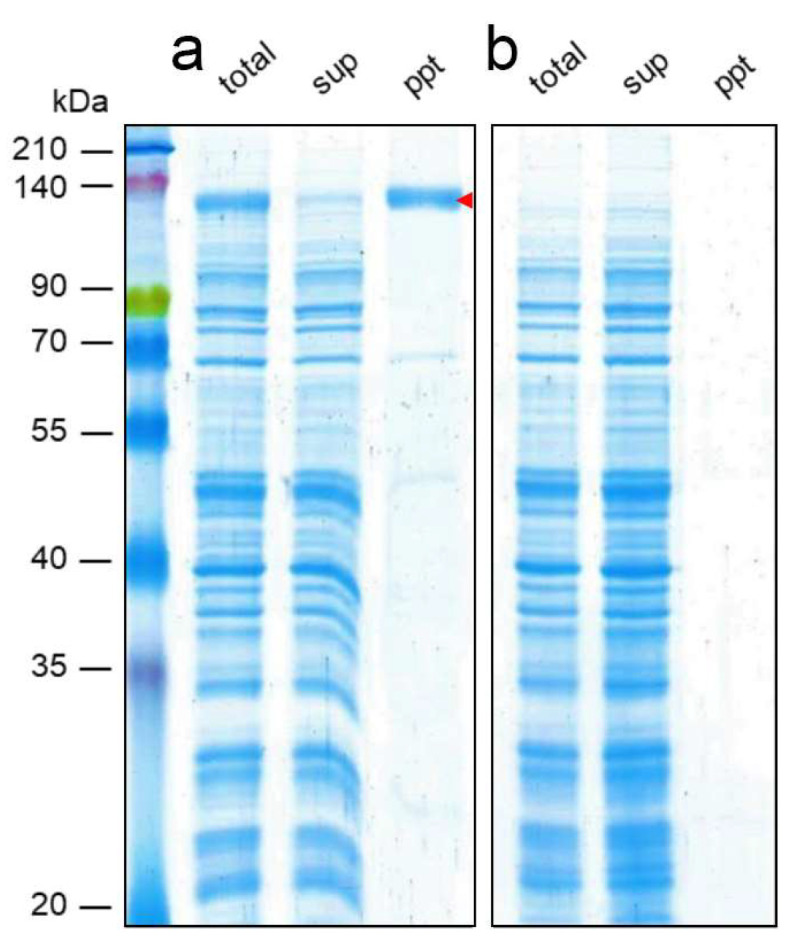
(**a**) hERG channel synthesized using a wheat germ cell-free translation system in the presence of PC+PE+Chol-vesicle, separated by SDS-PAGE and stained with CBB. The red arrowhead indicates the band of the hERG channel. (**b**) PC+PE+Chol-vesicle treated with the wheat germ cell-free translation system without mRNA. sup: supernatant, ppt: precipitate.

The preparation of PLs incorporating the hERG channel followed the cell-free synthesis system that was previously described [26]. In brief, the recombinant hERG channel protein (UniProt/SWISS-PROT accession no. Q12809), encoded in the human genome, was produced using a wheat germ cell-free translation system in the presence of the PC+PE+Chol vesicles. Afterward, the mixtures were centrifuged at 15,000× *g* for 15 min at 4 °C. The precipitate in the pellet contained the hERG channel–liposome complex and was suspended in a buffer solution (100 mM potassium acetate and 30 mM HEPES/KOH [pH 7.8]), with the same volume as the translation reaction mixture. This suspension underwent another centrifugation at 15,000× *g* for 15 min at 4 °C, and the resulting precipitate was collected separately. This process was repeated twice, and the final precipitate was resuspended in the buffer solution and stored at −80 °C until used. For analysis, we examined 1/100 of the volume from each fraction using sodium dodecyl sulfate polyacrylamide gel electrophoresis (SDS-PAGE). Following SDS-PAGE, the gel underwent Coomassie Brilliant Blue (CBB) staining. Translational integration of the hERG channel monomer (127 kDa) into lipid vesicles was confirmed by the band detected in the precipitate fraction (Figure 1a) [44]. The band of the hERG channel did not appear when the PC+PE+Chol-vesicle was treated in the same way with the cell-free translation system that did not include mRNA (Figure 1b).

SLBs were prepared using the vesicle fusion method with PL, following a similar procedure as for PC+PE+Chol-SLB in previous studies [30,31,32]. The suspension of PL was extruded through a polycarbonate filter with 800 nm pores to prevent the adsorption of aggregated PLs. A freshly cleaved piece of muscovite mica was incubated in the suspension at 37 °C for 2 h. The suspension was exchanged with the PL-free buffer solution 15 times to remove excess PLs. For fluorescence observation, 5 µL of an ethanol solution of Bis-Bodipy-FL-PC (0.4 M) was added to the 400 µL of the buffer solution in the SLB sample cell. The SLB was incubated at 25 °C for 1 h in the presence of Bis-Bodipy-FL-PC, followed by washing with the buffer solution 15 times.

AFM and fluorescence observations of SLBs were performed in the buffer solution at 25 °C following the conditions outlined in previous studies [31,32]. AFM topographies were obtained with PicoPlus 5500 (Keysight Technologies, Inc., Santa Rosa, CA, USA, formerly Molecular Imaging, Corp.), in acoustic AC mode (intermittent contact mode). A Si_3_N_4_ cantilever with a spring constant of 0.1 N/m and a tip curvature radius of 8 nm (BL-AC40TS-C2, Olympus, Tokyo, Japan) was used. The area and height of oligomers in the AFM topographies were obtained using particle analysis of Scanning Probe Image Processor software v.6.7.9 (SPIP, Image Metrology A/S, Hørsholm, Denmark). The averaged image of hERG channel molecules in the AFM topographies was processed using Image J software (NIH, http://imagej.nih.gov/ij/, accessed on 31 July 2023). Observations of fluorescence microscopy and fluorescence recovery after photobleaching (FRAP) were conducted with an epifluorescence microscope (BX51WI, Olympus, Tokyo, Japan), equipped with a 100× water-immersion lens (LUMPlan FL 100×, NA = 1.00, Olympus) and the mirror unit U-MWB2 (Ex: 460–490 nm, Em > 520 nm, Olympus). Fluorescence images of Bis-Bodipy-FL-PC were recorded with a CMOS camera (DS-Qi2, Nikon, Tokyo, Japan). 

## 3. Results and Discussion

**Figure 2:**
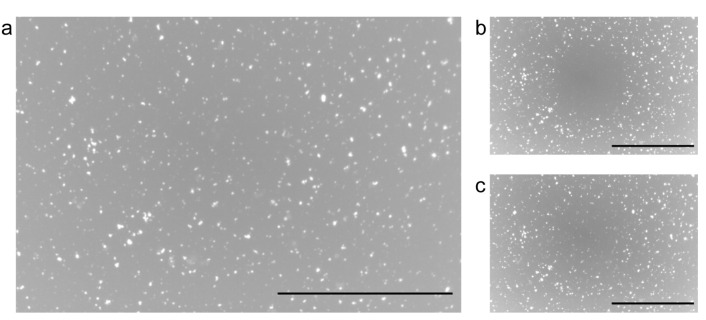
(**a**) A fluorescence image of the mica substrate after incubation in the PL suspension, followed by staining with Bis-Bodipy-FL-PC. (**b**,**c**) The FRAP process obtained at the same position as (**a**): (**b**) 0 s and (**c**) 300 s after the photobleaching. Images are presented in grayscale to facilitate visualization. Scale bars = 50 μm.

A single-layered SLB is formed on a mica substrate using PC+PE+Chol-vesicle with the same components as in this study [30,31,32]. The SLB made from the PC+PE+Chol-vesicle, including a dye-labeled lipid, exhibits uniform fluorescence intensity, and the unruptured PC+PE+Chol-vesicles are observed as bright spots in a fluorescence image (Figure S1a in Supporting Information). We first investigated the formation of the SLB using PL that contains the hERG channel in the PC+PE+Chol-vesicle. In the cell-free synthesis system, the hERG channel was expressed in PC+PE+Chol-vesicle without a fluorescence probe. Therefore, the fluorescence image of the SLB made from the PL was observed after the incorporation of Bis-Bodipy-FL-PC. Figure 2a shows a fluorescence image of the mica substrate after being incubated in the hERG-expressed PL suspension and stained with Bis-Bodipy-FL-PC. Uniform fluorescence intensity covered the entire surface of the substrate, accompanied by several bright spots. They correspond to the SLB and unruptured PLs, respectively, as seen in the fluorescence image of the PC+PE+Chol-SLB (Figure S1a in Supporting Information) [30,31,32]. Figure 2b,c shows the process of FRAP. Upon partial SLB photobleaching of the SLB (Figure 2b), the fluorescence intensity of the photobleached region recovered over time (Figure 2c), indicating the lateral diffusion of the dye molecules in SLB. These results reveal that a fluid and continuous full-coverage SLB was formed from PL that contains the hERG channel molecules.

**Figure 3:**
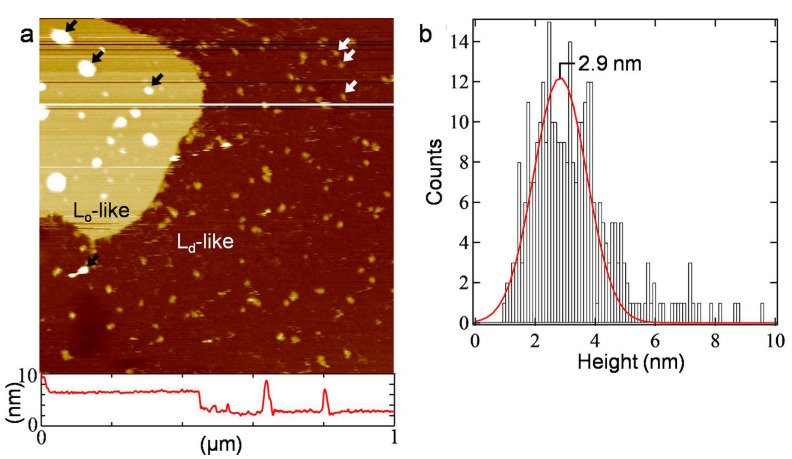
(**a**) An AFM topography (1.0 × 1.0 μm^2^) of the SLB prepared from PL using the same protocol as that of Figure 2 but without staining, along with a cross-section profile at the white line. Representative protrusions existing mainly in the L_d_-like and L_o_-like regions are indicated with the white and black arrows, respectively. (**b**) Histogram of the height of the protrusions existing in the L_d_-like region. The red curve represents the result of the Gaussian fitting.

The SLB prepared from the hERG-expressed PL was observed with AFM to investigate the hERG channel molecules and their association states in the bilayer (Figure 3). Two distinct regions with different heights were observed in the AFM topography (Figure 3a). This result is consistent with the previous studies [30,32], indicating phase separation in the PC+PE+Chol-SLB to the L_o_-like region, mainly comprising PC and Chol, and the L_d_-like region, which is rich in polyunsaturated PE [33]. The L_o_-like region is thicker than the L_d_-like region because of the ordering effect of Chol, and thus appears higher in AFM topography [33]. In the L_d_-like region, protrusions were observed (indicated by white arrows in Figure 3a) with a uniform height of approximately 3 nm, as shown in the histogram in Figure 3b. These protrusions did not exist in the SLB prepared from the PC+PE+Chol-vesicle without the hERG channel (Figure S1b in Supporting Information), consistent with the AFM topographies of the PC+PE+Chol-SLB in the previous studies [30,32]. Protrusions higher than 10 nm and with various lateral sizes were also present (Figure 3a, indicated by black arrows). They are attributed to unruptured PLs adsorbed on the substrate or SLB surface [45,46]. The majority of the SLB was occupied with the L_d_-like region. The L_o_-like domains were smaller than 1 μm, making them indistinguishable in the fluorescence image (Figure 2). The lipid composition of the SLB prepared from the hERG-expressed PL is not determined from the fluorescence image and AFM topography, but it is possibly different from that of the PC+PE+Chol-vesicle. 

The PC+PE+Chol-SLB exhibits a flat surface (Figure S1b in Supporting Information) [30,32], and the protrusions indicated by the white arrows in Figure 3a were observed after the expression of the hERG channel (Figure 1a). The protrusions in the L_d_-like region are assigned to the outer membrane region of the hERG channel. During the cell-free synthesis of membrane proteins in the presence of vesicles, the proteins are synthesized outside of the vesicles and reconstituted into the lipid bilayer. Consequently, the intracellular part of the membrane proteins faces the outside of the vesicle. The large outer membrane region of the hERG channel on the intracellular side, which includes the proximal N-terminus and distal C-terminus, exists outside the vesicles and prevents flip-flop once the hERG channel is reconstituted in the lipid bilayer. The unimodal distribution of the hERG channel height in the AFM topography (Figure 3b) indicates that the orientation of the hERG channel in the SLB is uniform. The height value in Figure 3 is reasonably close to the size of the outer membrane region of the hERG channel on the extracellular side [18]. The extracellular side of the hERG channel faces upward in the SLB, assuming an inside-up configuration in the SLB formed through adsorption, rupture, and spread of PLs on the substrate surface. Membrane proteins solubilized from cell membranes as micelles take a random orientation after reconstitution into a lipid bilayer using the detergent removal method [47]. The aligned orientation of membrane proteins in PL is an advantage of the cell-free synthesis system.

**Figure 4:**
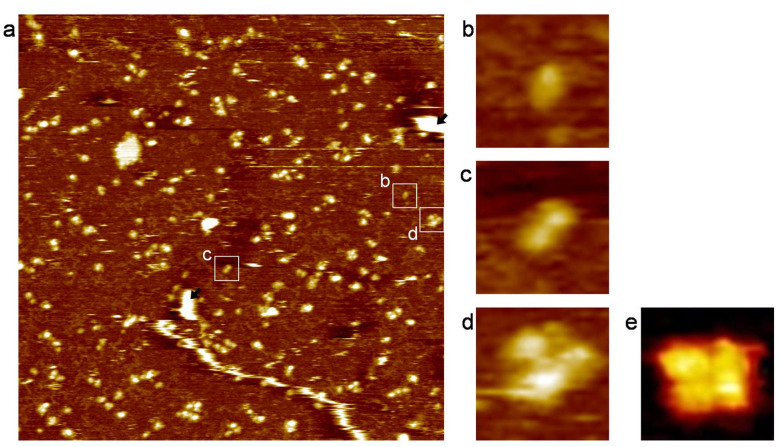
(**a**) An AFM topography (1.0 × 1.0 μm^2^) of the PC+PE+Chol-SLB including the hERG channel molecules showing their association states Unruptured PLs are indicated with the black arrows as those in Figure 3a. Magnified images (50 × 50 nm^2^) at (**b**–**d**) show representative (**b**) monomer, (**c**) dimer, and (**d**) tetramer structures. (**e**) An averaged image obtained from the AFM topographies of eight tetrameric hERG channel molecules.

Figure 4a shows molecular images of the hERG channels in the L_d_-like region of the PC+PE+Chol-SLB. A magnified image in Figure 4b reveals a representative single-blob structure, indicating the single subunit of the membrane-embedded hERG channel molecule. The hERG channel monomers are distinguished from the unruptured vesicles (Figure 4a, indicated by black arrows) based on their height and width as shown in Figure 3. Figure 4c,d reveals the association of two and four protrusions, respectively, with a similar height to that of the isolated protrusion (Figure 4b). These topographic images are attributed to dimers and tetramers of the hERG channel molecules, respectively. Figure 4e shows the averaged image obtained from eight individual tetrameric protrusions. The AFM topographies visualize each oligomeric state of the hERG channel molecules, including monomers, dimers, and tetramers. We also observed instances of three blobs linking together, but they did not always have a triangular state. It is worth noting that the hERG channel molecules did not exhibit diffusion within the SLB, consistent with the behavior observed in prior studies of membrane proteins embedded in SLBs [40,41,42,43]. The oligomeric states present in PLs were captured within the SLB.

**Figure 5:**
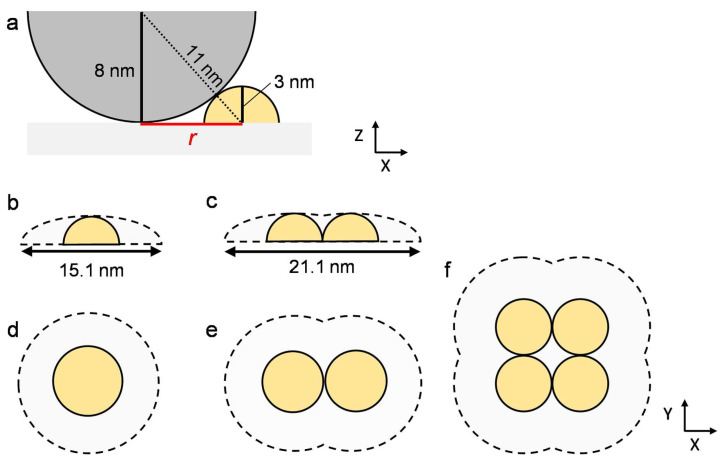
Schematics illustrating the effect of tip size on apparent lateral size in AFM topographies. (**a**) The cantilever tip (curvature radius = 8 nm) in contact with a 3 nm high hERG channel monomer assumed to be a hemisphere at a distance r. (**b**,**c**) Traces of the tip scanning (**b**) a monomer and (**c**) a dimmer or a tetramer. (**d**–**f**) Apparent areas of (**d**) a monomer, (**e**) a dimer, and (**f**) a tetramer including the effect of the tip size.

We evaluated the area of the hERG channel oligomers in the AFM topography, taking into consideration the tip size of the cantilever. The area of the hERG channel monomers, dimers, and tetramers obtained from the AFM topography was 190 ± 5, 285 ± 11, and 470 ± 39 nm^2^, respectively (Table 1). In AFM topographies, the lateral size of objects was overestimated because of the shape of the cantilever tip (Figure 5) [48]. The curvature radius of the tip in this study is 8 nm, as provided by the supplier (BL-AC40TS-C2, Olympus). We assumed the outer membrane region of the hERG channel to be a hemisphere with a radius of 3 nm based on the height in Figure 3b. The tip made contact with the hERG channel at the distance (*r* in Figure 5a) of 7.55 nm. Figure 5b shows the trace of the tip scanning a hERG channel monomer, resulting in an apparent diameter of 15.1 nm. The trace of the tip on a dimer and a tetramer (Figure 5c) provides their apparent width of 21.1 nm. The apparent areas of the hERG channel monomer (Figure 5d), dimer (Figure 5e), and tetramer (Figure 5f) were 180, 270, and 400 nm, respectively, and correspond well to the experimentally obtained area values (Table 1). The results support the hERG channel monomer forms specific association states in the SLB. 

As the association states of the hERG channel monomers are evaluated using the AFM molecular images, we also quantitatively assessed the efficiency of the oligomer formation. The number of the oligomers is summarized in Table S1 in Supporting Information. The percentage abundance decreases with the oligomeric order of the hERG channel, with monomers at 70%, dimers at 24%, trimers at 4%, and tetramers at 2% (*N* = 173). This result indicates that the hERG channels synthesized in the cell-free translation system predominantly exist as monomers and dimers. However, the tetrameric state is essential for the function of the hERG channel in forming the pore. A recent study demonstrated the channel activity of hERG channels expressed using the same cell-free synthesis system as in the present study [26]. Therefore, at least a portion of the hERG channel tetramers observed in the AFM topography retain channel functionality, but more than 98% of the expressed hERG channel molecules exist in inactive states. 

Then, we performed experiments aimed at promoting the tetrameric assembly of the hERG channel molecules. A specific protein family, known as J-domain-containing proteins (J-proteins) serves as co-chaperones for heat shock protein 70 (HSP70), functioning to enhance protein folding and prevent aggregation of client proteins. Proper protein folding can facilitate the assembly of channel subunits into stable complexes. Recent research has indicated that DNAJB12 and DNAJB14, which are both types of J-proteins, play roles in stabilizing hERG channel subunits and promoting their tetrameric assembly [49]. Therefore, to facilitate the tetrameric assembly of the hERG channel, we cloned and expressed human DNAJB12 in liposomes reconstituted with the hERG channel. 

**Figure 6:**
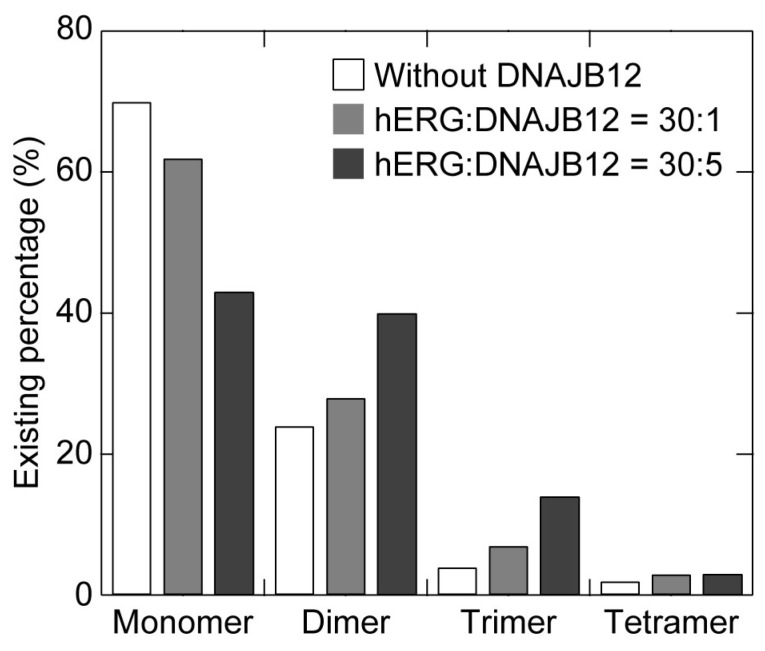
Existing percentage of hERG channel monomers in the forms of monomers, dimers, trimers, and tetramers and their dependence on the ratio of coexpressed DNAJB12. The coexpression ratios for hREG:DNAJB12 were 30:0 (white bars), 30:1 (gray bars), and 30:5 (black bars). Numerical values are summarized in Table 2.

We examined the effect of coexpression of DNAJB12 and its expression level on the oligomerization of the cell-free synthesized hERG channel monomer. The hERG channel and DNAJB12 were expressed at ratios of 30:1 and 30:5 in the presence of the PC+PE+Chol-vesicle to prepare PLs, and SLBs were prepared using the PLs. The percentage of existing hERG channel monomers in the forms of monomers, dimers, trimers, and tetramers is shown in Figure 6, with numerical values listed in Table 2. The existing ratio of the monomer decreased with the coexpression ratio of DNAJB12, while the ratio of oligomers increased. More dimers and trimers, corresponding to fewer monomers, appeared with the higher expression ratio of DNAJB12. The significant increase in the dimer percentage compared to that of tetramer suggests that DNAJB12 promoted the assembly of the hERG channel by facilitating its initial dimerization step. However, the coexpression of DNAJB12 did not significantly affect the existing ratio of the tetramer. It has been reported that knockdown of both DNAJB12 and DNAJB14 in human neuroblastoma SH-SY5Y cells significantly reduces the expression level and the channel current activity of the hERG channel, while knockdown of one of them has a partial effect [49]. DNAJB14 possibly play a role in the assembly processes from the dimer to the tetramer, and additional coexpression of DNAJB14 may enhance the efficiency of the tetrameric assembly in this study. Future research is needed to elucidate the mechanism of the tetrameric assembly of hERG channel molecules.

There are several challenges that need to be addressed to enhance the throughput of ion channel studies using artificial lipid bilayer systems. These challenges include the stability of the lipid bilayer, the reconstitution probability of the ion channel, and the efficiency of the ion channel expression [28,38,47,50]. Recently, Hirano-Iwata and colleagues developed a microfabricated silicon chip with a nanotapered aperture and a liquid chamber designed to apply centrifugal force for BLM experiments [26,28]. The nanotapered aperture enhances the robustness of the free-standing bilayers, while the centrifugal force accelerates the frequency of PL fusion into the BLM. PLs obtained from membrane crude extracts of Chinese hamster ovary (CHO) cell lines expressing the hERG channel achieve a success rate of 67% in the channel current recording [28]. In contrast, the hERG-expressed PL prepared with the cell-free synthesis system resulted in a success rate of 31% [26]. Success in the channel current recording implies that at least one active hERG channel tetramer in the hERG-expressed PLs is reconstituted to the BLM, accompanied by an abundance of inactive monomers and dimers. Enhancing oligomerization within the cell-free synthesis system is instrumental in improving the throughput of channel current recordings. The structural observations of hERG channel molecules in this study offer an analytical method for assessing oligomerization states at the molecular level, making it a valuable resource for future drug design efforts aimed at preventing drug-induced side effects on the hERG channel.

## 4. Conclusions

In this study, we investigated the association states of the cell-free synthesized hERG channel monomer using AFM. Molecular images of the hERG channel in the SLB allowed for a quantitative analysis of the oligomerization efficiency of the hERG channel monomers. The PL incorporating the full-length hERG channel with high purity was obtained after the cell-free synthesis of the hERG channel in the presence of PC+PE+Chol-vesicle. The SLB was prepared from the PL, and its AFM topographies showed protrusions with uniform height, indicating the aligned orientation of the hERG channel in the PLs and SLBs. These hERG channel monomers existed as either isolated states or specific associated states assigned to dimers and tetramers. The methods employed in this study are valuable for assessing and enhancing the expression efficiency of membrane proteins in their “active” form.

## Figures and Tables

**Table 1:** Average area values of the hERG channel oligomers obtained from the AFM topographies, along with the calculated values accounting for the size of the cantilever tip, as shown in Figure 5.

	Monomer	Dimer	Tetramer
Average area (nm^2^)	190 ± 5	285 ± 11	470 ± 39
Calculated value (nm^2^)	180	270	400

**Table 2:** Existing percentage of the hERG channel monomers in the forms of monomers, dimers, trimers, and tetramers in the absence and presence of coexpressed DNAJB12.

Expression Ratio (hERG:DNAJB12)	Monomer	Dimer	Trimer	Tetramer
30:0 *	70	24	4	2
30:1	62	28	7	3
30:5	43	40	14	3

* Without DNAJB12.

## Data Availability

Data is contained within the article and supplementary materials.

## Supplementary Materials

The following supporting information can be downloaded at: https://www.mdpi.com/article/10.3390/membranes13110854/s1, Figure S1: the morphology of the SLB made from PC+PE+Chol-vesicles without the hERG channel; Table S1: Number of the hERG channel oligomers.
