# Supplementary material for: Assembly of Cell-Free Synthesized Ion Channel Molecules in Artificial Lipid Bilayer Observed by Atomic Force Microscopy
Source: Membranes (Basel). 2023 Oct 25;13(11):854. doi: 10.3390/membranes13110854 (PMC10673230; doi:10.3390/membranes13110854)
Supplement: Supplementary file 1 [file membranes-13-00854-s001.zip › membranes-2587641-supplementary.pdf]

Supporting Information

Assembly of cell-free synthesized ion channel molecules in artificial lipid bilayer observed by atomic force microscopy

Melvin Wei Shern Goh [1], Yuzuru Tozawa [2] and Ryugo Tero [1,*]

[1] Department of Applied Chemistry and Life Science, Toyohashi University of Technology, Toyohashi, 441-8580, Japan; melvin.goh.wei.shern.dk@tut.jp (M. W. S. G)

[2] Graduate School of Science and Engineering, Saitama University, 255 Shimo-Okubo, Sakura-ku, Saitama, 338-8570, Japan; tozawa@mail.saitama-u.ac.jp

* Correspondence: tero@tut.jp

Morphology of SLB made from PC+PE+Chol-vesicles without hERG channel

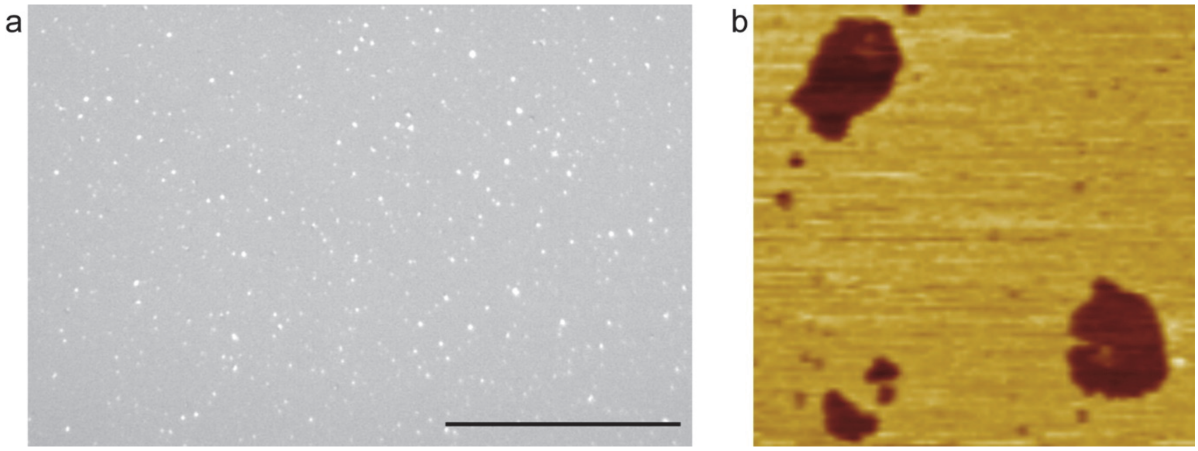

Figure S1. (a) A fluorescence image (Scale bars = 50 μm) and (b) an AFM topography (2.0 × 2.0 μm^2) of the SLB prepared from PC+PE+Chol-vesicles that did not include the hERG channel. The PC+PE+Chol-vesicles contained 0.26 mol% of a dye-labeled lipid, dipalmitoylphosphatidylethanolamine-*N*-lissamine rhodamine B.

Number of hERG channel oligomers

Table S1. The number of hERG channel oligomers observed in the AFM topographies of the PC+PE+Chol-SLB, including hERG channel molecules.

	Monomer	Dimer	Trimer	Tetramer
Number	122	41	7	3
